# ZD6474 attenuates TGF-β1-induced fibrosis in human Tenon fibroblasts and inhibits neovascularization via AKT-mTOR signaling pathway

**DOI:** 10.1007/s10792-022-02548-3

**Published:** 2022-10-13

**Authors:** Wenting Liu, Yaying Chen, Xiangyuan Song, Yiwen Xue, Yuyan Zhang

**Affiliations:** 1 Department of Ophthalmology, Huadong Hospital, Fudan University, No. 221 East Yan’an Road, Shanghai, 200031 China; 2 Department of Ophthalmology, Tongren Hospital, Shanghai Jiaotong University, Shanghai, China; 3 Department of Ophthalmology, Shanghai Pudong Hospital, Fudan University Pudong Medical Center, Shanghai, China; 4 Undergraduate School, Shanghai Medical College, Fudan University, Shanghai, China

**Keywords:** ZD6474, Anti-fibrosis, Anti-angiogenesis, Glaucoma surgery

## Abstract

**Purpose:**

To investigate the anti-fibrotic effect of ZD6474 (a novel inhibitor of VEGF and EGF) in TGF-β1 stimulated human Tenon’s capsule fibroblasts (HTFs) and the anti-angiogenetic role in HUVECs, compared to that of mitomycin C (MMC).

**Methods:**

The effects of ZD6474 on cell proliferation or migration in TGF-β1-stimulated HTFs and HUVECs were determined, using CCK8 or wound healing assay, respectively. The typical markers of fibrosis in TGF-β1-stimuated HTFs were detected, vimentin by immunofluorescence, α-SMA and snail by western blot. Tube formation was applied to validate the anti-angiogenesis effect in HUVECs following ZD6474 treatment. Furthermore, phosphorylated AKT and mTOR (p-AKT and p-mTOR) were evaluated, compared to the standardized total AKT and mTOR, using western blot.

**Results:**

There was almost no decreased cell viability in HTFs following ZD6474 (≤ 1 μM/mL) treatment, but MMC (> 50 μg/mL) significantly impaired cell viability. ZD6474 significantly inhibited TGF-β1-stimulated proliferation and migration in HTFs, compared to control group (**P < 0.01). ZD6474 also significantly attenuated the TGF-β1-stimulated expression of vimentin, α-SMA and snail in HTFs. Tube formation was notably interrupted in HUVECs following ZD6474 treatment (**P < 0.01). P-AKT and p-mTOR were significantly decreased in response to ZD6474 treatment in TGF-β1- induced HTFs and HUVECs.

**Conclusions:**

ZD6474 exerts anti-proliferation and anti-fibrotic effects in TGF-β1-stimulated HTFs perhaps via regulating AKT-mTOR signaling pathway. ZD6474 also inhibited proliferation, migration and tube formation in HUVECs via the same signaling pathway. We concluded that ZD6474 may be potentially a novel agent in preventing bleb dysfunction following glaucoma filtration surgery (GFS).

## Introduction

Glaucoma is the second leading cause of blindness affects millions of people. The global prevalence of glaucoma is 3.54% in the population aged 40–80 years old, with estimation of affecting 76 million worldwide in 2020 [1, 2]. Glaucoma filtration surgery (GFS) is the most effective treatment in the management of intraocular pressure (IOP) when medication and laser are insufficient. Although GFS is considered as an effective treatment, post-operative filtering bleb scar formation often leads to surgical failure [3, 4]. The formation of hypertrophic scars (HS) and excessive wound healing processes at the filtering bleb serve as the major failure following GFS [5, 6]. Fibrosis is a key during the progression of HS, which is closely associated with secretion of EGF and/or TGF-β [7, 8].

Neovascularization also plays an important role in the failure of GFS. VEGF is one of the hotspots in ocular diseases, especially in the field of neovascular fundus diseases and glaucoma [9, 10]. Some studies report that VEGF is notably upregulated following GFS at the early stage [11, 12]. Extensive studies have been explored in preventing GFS failure, either via developing potential new therapeutic agents and/or improving surgical techniques [13, 14]. Although anti-metabolites such as mitomycin-C (MMC) and 5-flurouracil (5-FU) can effectively reduce post-surgical scarring, it is not satisfactory for the serious adverse devastating and potentially sight‑threatening complications, such as bleb leak, hypotony and corneal toxicity [15, 16]. Consequently, it is extensively explored that the potential therapeutic agent(s) with high anti-scar formation, but low or no cytotoxicity.

**Fig. 1:**
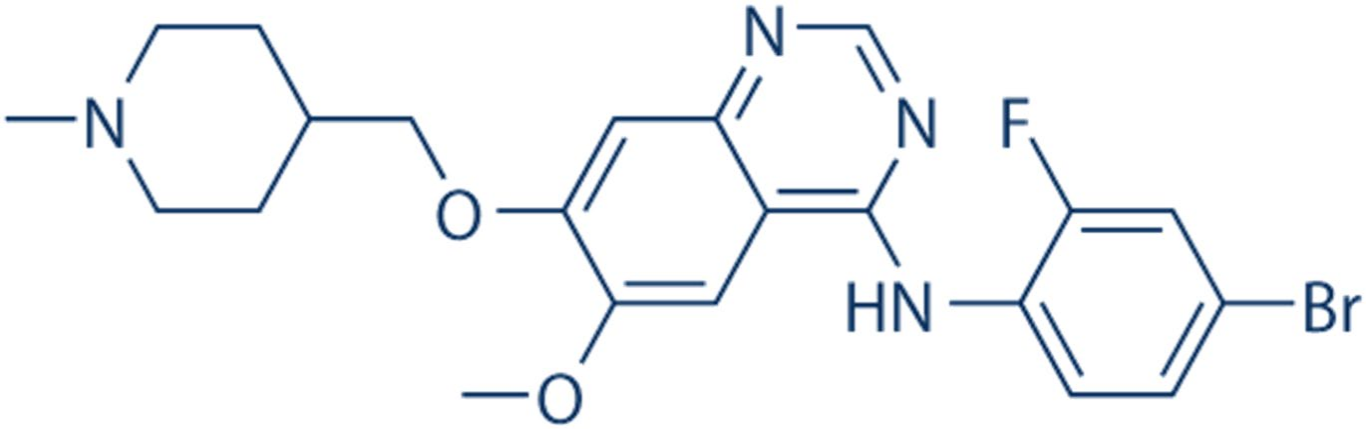
Chemical structure of ZD6474

ZD6474, a compound with molecular weight of 475 Da, is a tyrosine kinase inhibitor (TKI) with an affinity to multiple growth factor receptors, including VEGFR-2 (IC50 40 nM), VEGFR-3 (IC50 110 nM) and EGFR (IC50 500 nM) (Fig. 1) (Beyotime Institute of Biotechnology, Shanghai, China). ZD6474 has been approved for the treatment of patients with thyroid cancer [17]. [FDA Center for Drug Evaluation and Research. Labeling-Package Insert. Available online: https://www.accessdata.fda.gov/ drugs at FDA docs/label/2020/022405s017lbl.pdf (accessed on 30 January 2021)]. ZD6474, a co-inhibiter of VEGFR/EGFR and mTOR kinases, is also illustrated substantially higher anti-proliferative activity than either VEGFR inhibitor or EGFR inhibitor alone applied in cancers [18]. Due to multi-kinase inhibitors can accurately act on multiple signal pathways and their cascade, so it can achieve a wide range of therapeutic effect. Therefore, we speculated that ZD6474 (multi-kinase inhibitor) can similarly exert biological functions in minimizing fibrosis and/or neovascularization following GFS, which would be a good candidate in the management of potential adverse situation post-surgery (GFS).

The aim of this study was to investigate anti-fibrosis effect of ZD6474 in TGF‑β1‑ stimulated HTFs in vitro. In addition, the anti-angiogenesis effect of ZD6474 in HUVECs was also evaluated. These findings may aid to develop ZD6474 as a novel agent for the prevention of GFS failure.

## Materials and methods

### HTFs culture and morphology

The current study has been approved by the Institutional Review Board (IRB) of the Huadong Hospital, Fudan University. This study was conducted in accordance with the tenets of the Declaration of Helsinki. Written informed consent was obtained from every patient after explicit explanation of the study procedure and its possible benefits and risks. All human Tenon’s fibroblast samples were collected from these patients with consents. HTFs were obtained from three patients undergoing cataract surgery from June 2020 to Oct 2020 with the following criteria: no ocular disease except cataract, no prior history of ocular surgery and trauma.

Human Tenon’s fibroblast tissues were excised from patients during surgery, and these tissues were propagated in DMEM (Hyclone Life Technologies, USA) supplemented with 10% FBS (Gibco Life Technologies), 100 U/mL penicillin and 100 g/mL streptomycin (Gibco Life Technologies). Subsequently, the cells were maintained at 37 °C in 5% CO_2_ in a humidified condition, and the medium was changed every 3 days thereafter. HTFs between the third and eighth passages were used for following assays. Morphology of HTFs was photographed from the cells with 80% confluence in complete DMEM, using an upright microscope (Zeiss, Inc., Thornwood, NY, USA).

STR HUVECs which were purchased from Fuheng Bio-company were cultivated in Endothelial cell medium (ECM, Sciencell 1001, CA, USA) supplemented with 10% FBS (Gibco), 100 U/mL penicillin and 100 g/mL streptomycin (Gibco). Subsequently, cultivation procedures were as same as HTFs cultivation.

### Cytotoxicity and cell proliferation assay

Cytotoxicity assay was detected, using Cell Counting kit‑8 (CCK‑8; Dojindo Molecular Technologies, Inc., Kumamoto, Japan) according to the manufacturer's instructions. Cells under logarithmic phase were seeded (1 × 10^4^ cells/well) in 96-well plates with FBS-free medium for 24 h. These cells were treated with the various concentrations of ZD6474 (100 nM, 500 nM, 1 μM, 5 μM or 10 μM/ml) at 37 °C for 24 h after pre-hungry. To emulate clinical treatment, MMC (10 μg, 50 μg or 200 μg/ml) (Yuanchuang, Inc., Shanghai, China) was also added in these cells for 5 min then replaced with FBS free medium. Following 24 h treatment, all the media in the well were discarded and 10 μl CCK‑8 solution was added at 37 °C incubation for 1.5 h. The colorimetric absorbance was recorded at 450 nm, using a microplate reader (Bio‑Rad Laboratories, Inc., Hercules, CA, USA).

To investigate the effect of ZD6474 on post-operative scarring, we mimicked TGF‐β secretion by stimulating HTFs with TGF‐β1. Cells proliferation assay of HTFs and HUVECs were seeded at appropriate density in 96-well plates for assessment, using CCK-8 kit. To ascertain whether TGF‑β1 affects HTFs proliferation, HTFs were incubated ± TGF‑β1 (10 ng/ml) for 24, 48 or 72 h, respectively. Subsequently, the proliferation assay in HTFs was determined following treatment of ZD6474 (500 nM or 1 μM/ml) in presence with TGF-β1(10 ng/ml) for 24, 48 and 72 h and MMC 50 μg/ml for 5 min. In HUVECs, the proliferation assay was also determined with treatment of ZD6474 (500 nM or 1 μM/ml) for 24, 48 and 72 h and MMC 50 μg/ml for 5 min. After all exposure, 10 μl CCK8 was added in each well and incubated for 1.5 h. Finally, the colorimetric absorbance was also recorded at 450 nm to draw the proliferation curve.

### Wound healing assay

HTFs and HUVECs were incubated till 100% confluence and the cell monolayer was scratched with a 200 μl pipette tip. The scratched area was photographed by phase‑contrast microscopy (magnification, × 4). HTFs were added with fresh DMEM containing TGF‑β1(10 ng/ml) with or TGF‑β1 combined with ZD6474 (500 nM or 1 μM/ml) for 24 or 48 h, while 50 ug/ml MMC was added for only 5 min. HUVECs were added ZD6474 (500 nM or 1 μM/ml) in DMEM for 24 h, while 50 μg/ml MMC was only added for 5 min. Following different treatments, the distance of cell migration was recorded by phase‑contrast microscopy (magnification, × 4) and measured by Image J.

### Immunofluorescence staining

Immunofluorescence (IF) staining was applied for identification of HTFs and monitoring phenotype changes in fibroblasts. The trans-differentiation of fibroblasts into myofibroblasts is considered a pivotal step during fibrosis. At the molecular level, this process is characterized by the upregulation of some protein expressions, including vimentin, TGF‑β1 and α-SMA [19]. The slides covered with HTFS were incubated with anti-cytokeratin (1:200, Proteintech, Inc.cat No 24789–1-AP), anti-vimentin (1:200, Proteintech, Inc.cat No 60330–1-Ig) in a wet chamber at 4 °C overnight, and then were further incubated with goat polyclonal secondary antibody to mouse or rabbit (1:100, Beyotime, Inc.) in a light proof box after 3 times washing in TBS. Finally, the slides were labelled with DAPI (Beyotime, Inc.) for 3 min and coverslip. The labelled slides were observed under fluorescence microscopy (Leica DFC 310 FX, German).

### Tube formation assay

Since HUVECs have been identified as a well-established model for angiogenesis study in vitro, we further conducted the tube formation assay to investigate the anti-angiogenetic impacts of ZD6474 in HUVECs. Firstly, HUVECs (P3-P8) were treated with ZD6474 (500 nM or 1 μM/ml) for 24 h. Meanwhile, a 48-well ice-cold plate was coated with 250 μl/well Matrigel (BD, Bioscience, USA, 1:4 dilution in serum-free DMEM) and incubated for 30 min at 37 °C. After treatment, HUVECs in conditioned medium were overlaid on the Matrigel. Finally, the number of new capillary formation was observed under a microscope (Zeiss, Inc., Thornwood, NY, USA) and counted with Image J.

### Western blot

For mechanistic determination, the classic AKT-mTOR signaling pathway was evaluated, using western blot. HTFs and HUVECs with appropriate density were seeded in 6-well plates for 2 days. HTFs were treated with ZD6474 (500 nM or 1 μM/ml) and/or MMC combined with 10 ng/ml TGF-β1, while HUVECs were treated with ZD6474 (500 nM or 1 μM/ml) and/or MMC without TGF-β1. Total protein samples were isolated from the HTFs and HUVECs, using RIPA lysis buffer (Thermo Fisher Scientific, Inc.) supplemented with phosphatase inhibitors (Thermo Fisher Scientific, Inc.) and protease inhibitors (Thermo Fisher Scientific, Inc.). The protein samples were immediately stored at − 80 °C until use after quantity of protein was measured using BCA protein kit (Beyotime, Inc.).

Protein samples (10 μg) were separated via SDS‑PAGE with 8% of separation gel and 5% stacking gel, and then proteins were transferred to a polyvinylidene fluoride membrane (PVDF membrane, Millipore Corp., Bedford, MA, USA). Protein transferred PVDF membranes were blocked with 5% bovine serum albumin (BSA) and incubated with primary antibodies, including GAPDH (1:10,000, Proteintech, Inc.cat No 60004–1–lg), AKT rabbit polyclonal antibody (1:5000, Proteintech, Inc. cat No 10176–2–AP), phospho-AKT(Ser473) polyclonal antibody (1:5000, Proteintech, Inc.cat No 28731–1–AP), mTOR mouse monoclonal antibody (1:10,000, Proteintech, Inc. cat No 66888–1–lg), phosphor-mTOR mouse monoclonal antibody (1:5000, Proteintech, Inc. cat No 67778–1–lg), smooth muscle actin polyclonal antibody (α-SMA, 1:5000, Proteintech, Inc. cat No 55135–1–AP) and rabbit anti-SNAIL polyclonal antibody (1:1000, Bioss Institute of Biotechnology, Beijing, China) on the shaker at 4 °C overnight. The PVDF membranes with targeted protein were incubated with HRP-labeled goat anti-mouse/rabbit antibodies (1:1000, Beyotime, Inc.) for 1 h at room temperature. Immunoreactive bands were visualized using an enhanced chemiluminescence (Beyotime, Inc.), and the density of protein bands was captured by a GS-700 Imaging Densitometer (BIO-RAD, Hercules, CA, USA) and analyzed by Image J.

## Statistical analysis

Data were analyzed using SPSS V17.0 software (SPSS Inc.; Chicago, USA) and GraphPad Prism version 7.0 (GraphPad Software, Inc., La Jolla, CA, USA). Numerical data were presented as mean ± standard deviation (SD). Comparisons among multiple groups were performed by one‑way analysis of variance (ANOVA) with least-significant difference (LSD) post‑hoc test. The results are indicated as *P* values, where *P < 0.05 was considered to indicate a statistically significant difference. The normal distribution test was conducted to check whether the numerical variables were normally distributed.

## Results

### Cell morphology and expression levels of α-SMA and snail in HTFs

**Fig. 2:**
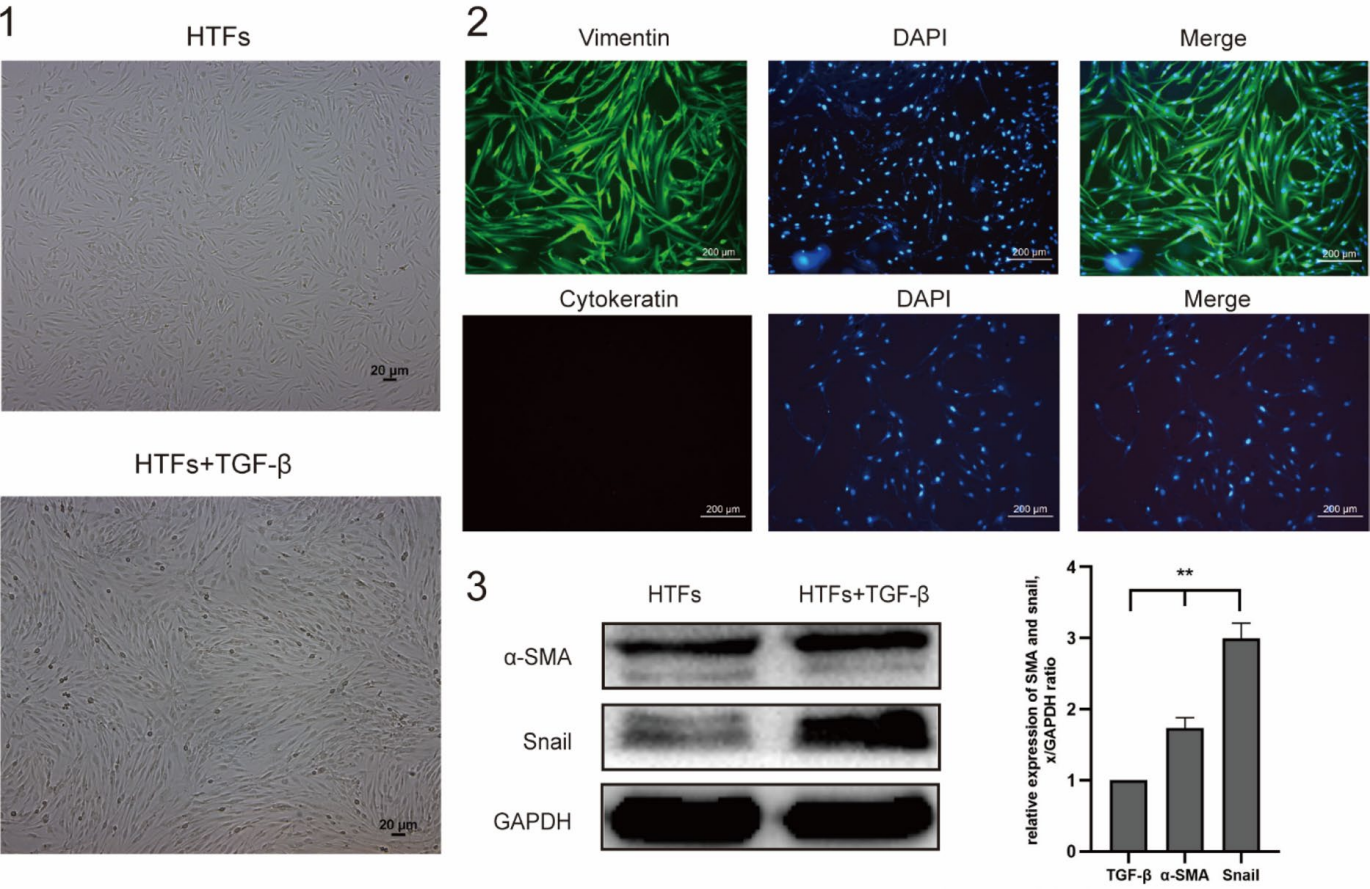
Morphology and identification of HTFs. **a** Morphology of HTFs ± TGF-β1 stimulation. **b** Fibroblast marker vimentin was stained but not epithelial cell marker cytokeratin in primary HTFs. Nuclei were labeled with DAPI. Scale bar 200 μm. **c** Representative western blot images of α-SMA and snail protein levels in HTFs with or without TGF-β1-stimulaiton, which was normalized to GAPDH as the loading control

The morphology for the primary HTFs exhibited a typical spindly and elongated shape with distribution similar to feather and vortex. HTFs proliferated rapidly and became more closely connected, following TGF-β1 stimulation (10 ng/ml) (Fig. 2a). Immunofluorescence staining revealed that vimentin, but not cytokeratin was extensively expressed in HTFs (Fig. 2b). Compared with untreated HTFs, α-SMA and snail were significantly increased in TGF-β1-stimulated HTFs indicated that TGF‑β1 can successfully trigger the conversion of fibroblasts into myofibroblasts in HTFs (Fig. 2c).

### Cytotoxicity and cell proliferation of ZD6474 on HTFs and HUVECs

**Fig. 3:**
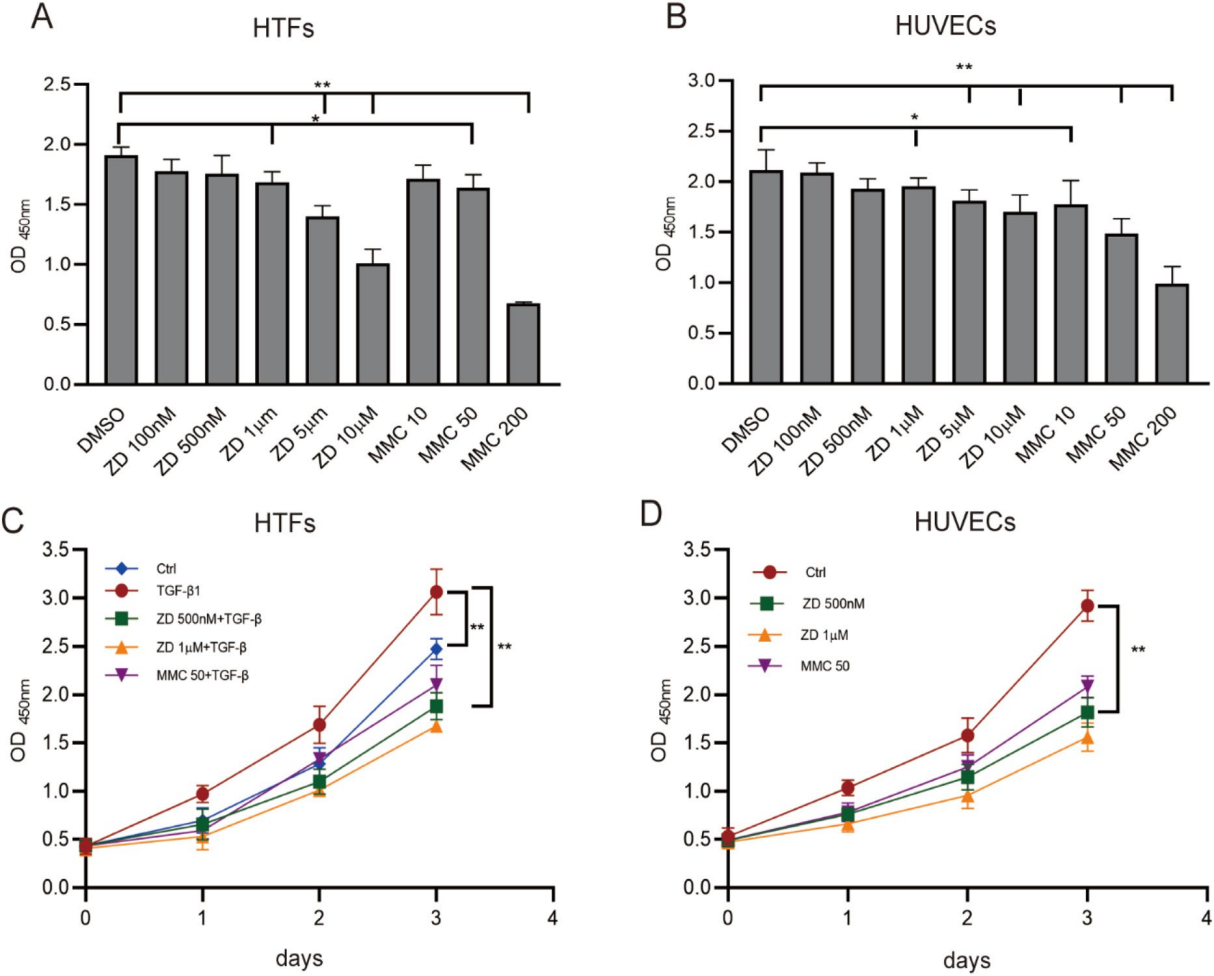
The effects of ZD6474 and MMC on cell viability and proliferation. **a** HTFs were exposed to different concentrations of ZD6474 (100 nM, 500 nM, 1 μM, 5 μM or 10 μM/ml) for 24 h and MMC (10, 50 or 200 ug) for 5 min. **b** HUVECs were exposed to different concentrations of ZD6474 (100 nM, 500 nM, 1 μM, 5 μM or 10 μM/ml) for 24 h and MMC (10, 50 or 200 μg) for 5 min. **c** Proliferation curve of HTFs exposed to control medium, medium with 10 ng/ml TGF‑β1 and 10 ng/ml TGF‑β1 in presence with ZD6474 (500 nM or 1 μM/ml) for 24, 48 and 72 h and MMC (50 μg/ml) for 5 min. **d** Proliferation curve of HUVECs exposed to ZD6474 (500 nM or 1 μM/ml) for 24, 48 and 72 h and MMC (50 g/ml) for 5 min. Data are expressed as percentage of each value. Error bars represent SD (*P < 0.05, **P < 0.01)

It was observed that MMC (> 50 μg/mL) reduced cell viability significantly in HTFs and HUVECs, compared that to the mock treated (**P < 0.01, Figs. 3). No significant difference of cell viability was observed between ZD6474 treated (< 1 μM/ml) and the mock treated group (Fig. 3a). Similar cytotoxicity results were also obtained in HUVECs (Fig. 3b).

Proliferation assay verified that TGF‑β1 (10 ng/ml) promoted HTFs proliferation significantly (3.063 ± 0.24 vs 2.471 ± 0.11, **P < 0.01) (Fig. 3c), but the effect was almost diminished in the presence of ZD6474 of 500 nM treatment for 72 h (3.063 ± 0.24 vs 1.879 ± 0.14, **P < 0.01, Fig. 3c). In HUVECs, cell proliferation was also mainly abated in the presence of 500 nM ZD6474 treatment for 72 h compared with the mock treated group (2.922 ± 0.16 vs 1.815 ± 0.16, **P < 0.01, Fig. 3d).

## ZD6474 inhibits migration in TGF‑β1‑induced HTFs and HUVECs

**Fig. 4:**
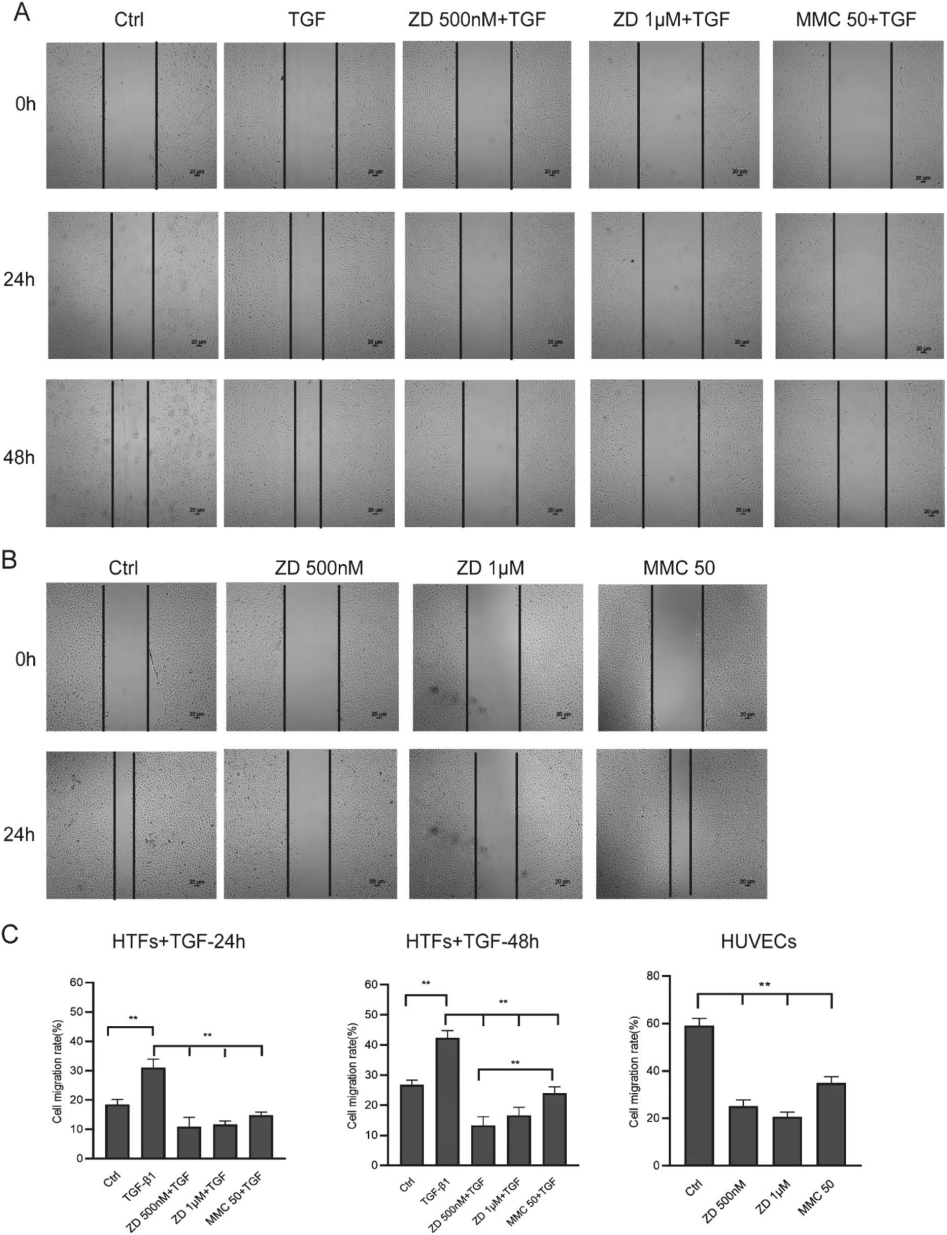
ZD6474 inhibits cell migration in TGF-β1-stimulated HTFs and HUVECs. Cell migration was evaluated by wound‑healing assay. **a** Representative images of the different treatment groups in TGF-β1-induced HTFs at 24 and 48 h after scratch. **b** Representative images of different treatment groups in HUVECs at 24 h after scratch. **c** Quantitative analyses of denuded area percentages (denuded area at the specified time point/denuded area at 0 h) after different treatments at different times in TGF-β1-induced HTFs and HUVECs. Data are presented as the means ± standard deviation of 3 independent repeats. *P < 0.05, **P < 0.01, vs. the TGF‑β1 or control group by one-way ANOVA followed by LSD test. Original magnification, × 4

Wound healing assay was conducted to evaluate the migratory activity of the cells in vitro, which is a part of the scarring process. TGF-β1 robustly induced the migration of HTFs, but in the presence of ZD6474 obviously attenuated the migration of HTFs stimulated by TGF-β1. Co-cultivated with TGF-β1 for 24 h, the cell migration area of 500 nM ZD6474 was about 10.87 ± 3.21%, much narrower than the area of untreated group (59.06 ± 3.13%, **P < 0.01, Fig. 4a and c).

Similar results were obtained in 48 h treatment. Co-cultivated with TGF-β1 for 48 h, the cell migration area of 500 nM ZD6474 was about 13.26 ± 2.91%, much narrower than the area of untreated group (42.50 ± 2.26%, **P < 0.01). MMC also attenuated the migration area (23.92 ± 2.22%) in TGF-β1-induced HTFs, but the migration area was slight wider than the area in ZD 500 nM (13.26 ± 2.91%, **P < 0.01; Fig. 4a and c).

In HUVECs, cell migration was also observed inhibited in the presence of ZD6474. The migration area in control group was 59.06 ± 3.13%, obvious wider than that of 500 nM ZD6474 (25.17 ± 2.60%, **P < 0.01; Fig. 4b and c). Generally, ZD6474 noticeably attenuated the migration of TGF-β1-induced HTFs and HUVECs.

### ZD6474 attenuated trans-differentiation to myofibroblasts in TGF-β1-induced HTFs

To ascertain conversion of fibroblasts into myofibroblasts, the typical markers of fibrosis and EMT were examined, including vimentin, α-SMA, and snail. Change in vimentin expression was evaluated using IF, while changes in α-SMA and snail were evaluated using western blot in TGF-β1- induced HTFs.

**Fig. 5:**
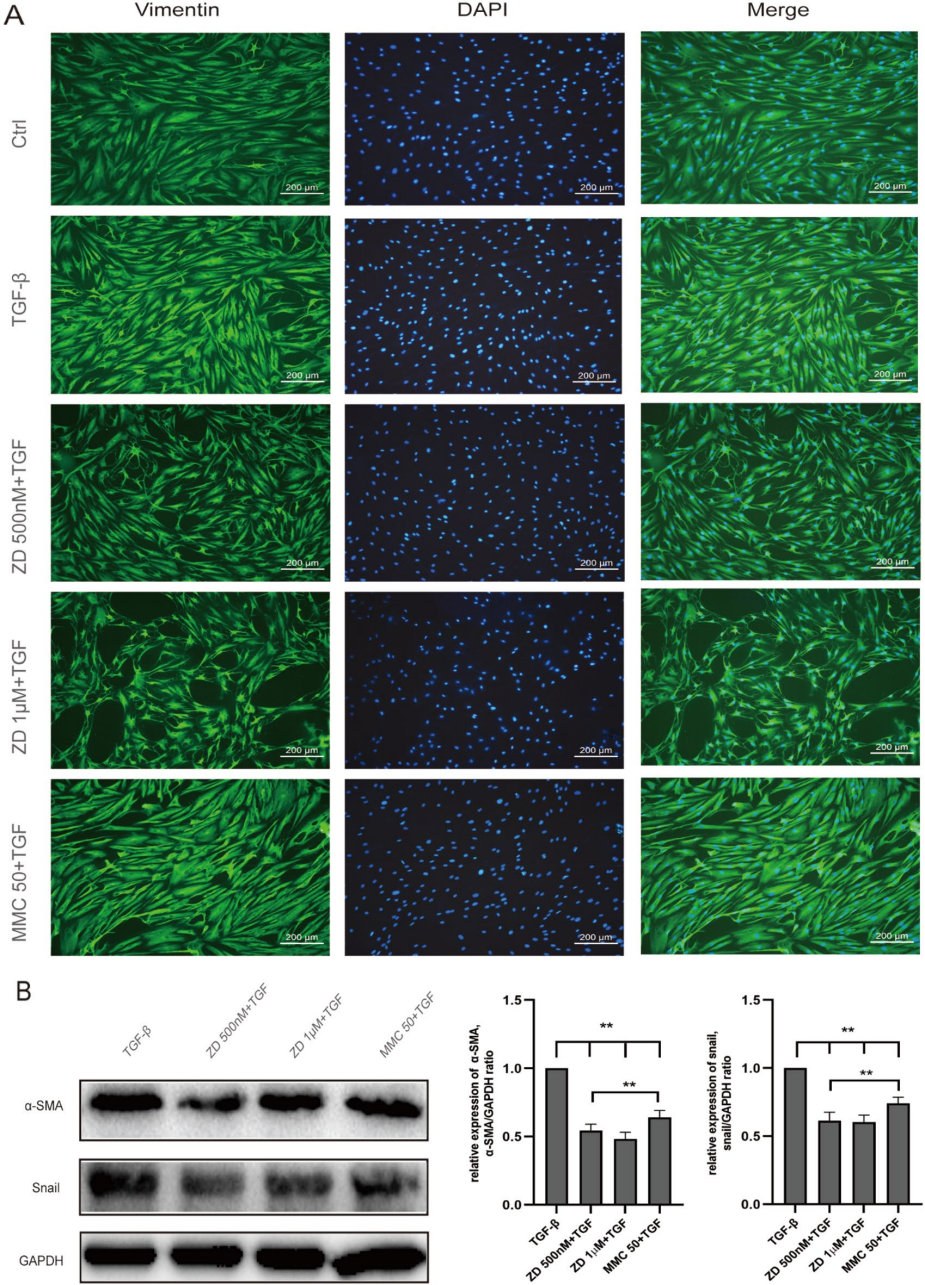
ZD6474 attenuated trans-differentiation to myofibroblasts in TGF-β1-induced HTFs. **a** Immunofluorescence was performed to detect vimentin (green) expression. The nuclei (blue) were labeled with DAPI. Scale bar, 200 μm. **b** Protein levels of snail and α-SMA in TGF-β1-induced HTFs were analyzed by western blot analysis and normalized to GAPDH expression

TGF‑β1 (10 ng/ml) significantly increased α-SMA, and snail protein expressions in HTFs indicated that TGF‑β1 can successfully trigger the conversion of fibroblasts into myofibroblasts in HTFs (Fig. 2c). ZD6474 (500 nM or 1 μM/ml) largely attenuated α-SMA, and snail expression in TGF‑β1-induced HTFs, which certified that ZD6474 can obviously inhibit the conversion progress of fibrosis in TGF-β1 activated HTFs (Fig. 5a and b).

### ZD6474 inhibits tube formation in HUVECs

**Fig. 6:**
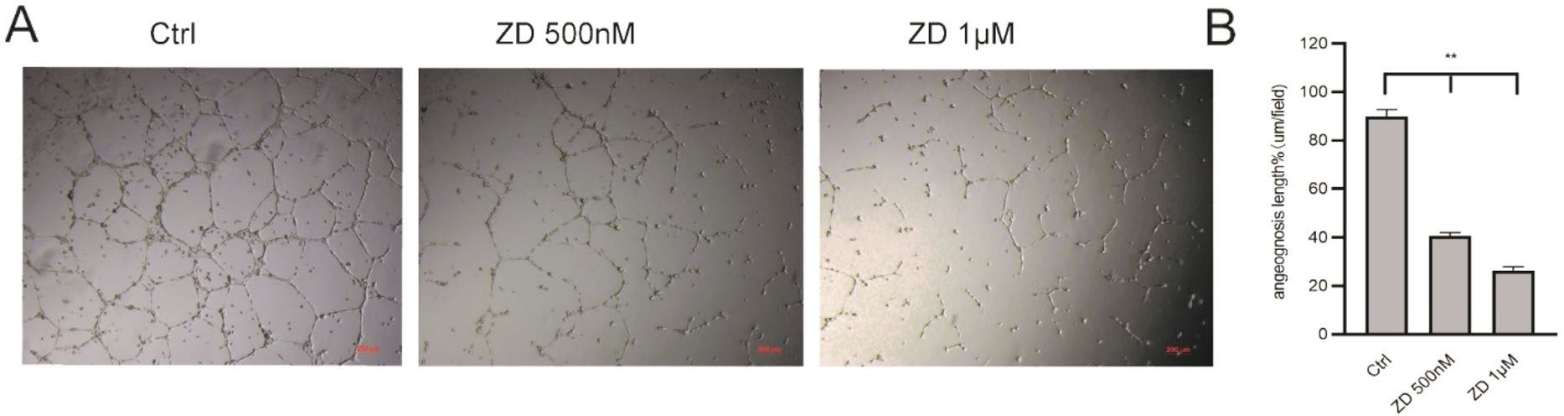
ZD6474 inhibits tube formation in HUVECs. Assessment of angiogenesis ability of HUVECs with or without ZD6474. **a** Tube and network formation assay of HUVECs treated with conditional media, 500 nM or 1 μM/ml ZD6474. **b** Branch points of capillaries were counted and analyzed. Scale bar = 100 μm. Data were presented as mean ± SD. *P < 0.05, **P < 0.01

Untreated HUVECS largely formed complete tubulars on the Matrigel. The angiogenesis length in untreated group was about 91.2%, while that of ZD6474 (500 nM or 1 μM) groups were much less than 50%. The tubule formation experiment indicated that the tubulars number in the ZD group were much lower than that in the untreated group (**P < 0.01, Fig. 6a and b).

### ZD6474 suppresses AKT-mTOR signal pathway in TGF‑β1-induced HTFs and HUVECs

**Fig. 7:**
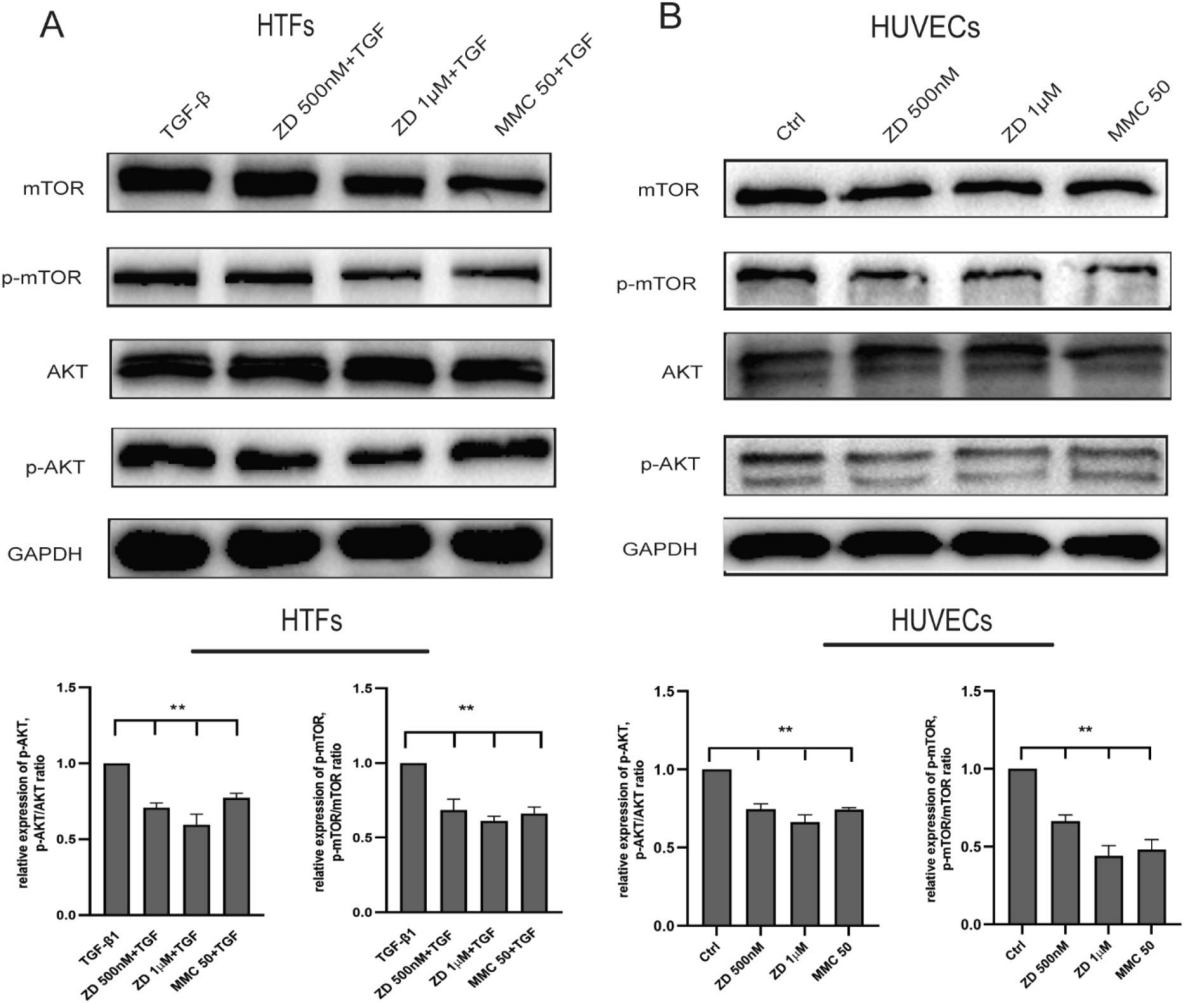
ZD6474 downregulated AKT-mTOR signal pathway in TGF‑β1-stimulated HTFs and HUVECs. **a** The expression levels of p-AKT and p-mTOR in TGF‑β1- stimulated HTFs were detected by western blot analysis and normalized to total AKT, mTOR and GAPDH, respectively. **b** The expression levels of p-AKT and p-mTOR in HUVECs were detected by western blot analysis and normalized to total AKT, mTOR and GAPDH. HTFs, human Tenon fibroblasts, HUVECs, human umbilical vein endothelial cells

AKT and mTOR phosphorylation (p-AKT and p-mTOR) protein levels were detected, using western blot and normalized to total AKT and mTOR proteins. In TGF‑β1-stimulated HTFs, western blot analysis showed that the expression levels of p-AKT and p-mTOR in the ZD group were significantly decreased (all **P < 0.01, Fig. 7a), whereas in HUVECs those proteins in the ZD group were significantly decreased too (all **P < 0.01; Fig. 7b). The results suggested that ZD6474 might play its biological function through downregulating the PI3k-AKT-mTOR signaling pathway.

## Discussion

Our study confirmed that ZD6474 reduced cell proliferation and migration in TGF-β1-stimulated HTFs. Trans-differentiation to myofibroblasts and EMT were also attenuated in presence with ZD6474 in TGF-β1-stimulated HTFs. In contrast with the obvious toxicity of MMC [20, 21], ZD6474 showed a selective toxicity effect on the normal HTFs. Hence, we identified the anti-fibrosis effect of ZD6474 in preventing scar formation following GFS with better safety than MMC.

GFS is to deliberately open an extra aqueous outflow pathway in trabecular meshwork to decrease IOP effectively, but there are still numerous potential complications post-operatively. After GFS, the re-blockage of the pathway due to scar formation (severe fibroblasts proliferation, extra ECM accumulation), resulting in upregulating IOP again is very common [22]. During the early stage of wound healing post-operatively, excessive TGF‐β promotes fibrosis perhaps via recruiting fibroblasts and increase fibroblast proliferation [23]. It has been reported that failure of GSF is mainly due to scar formation and extra ECM accumulation, particular in the presence of TGF‑β1 secreted by activated fibroblasts [24]. We found that stimulated with TGF‑β1 significantly promoted HTFs proliferation and migration, but these effects were largely diminished in presence with ZD6474, suggesting that ZD6474 is capable of reducing scar formation following GFS. Thus, ZD6474 (a multi-kinase inhibitor) can also target to both VEGF and EGF pathways, which are able to achieve similar therapeutic effects as bevacizumab and 5-FU. Hence, such finding suggests that ZD6474 may also exert equal therapeutic effect, but substantially reduce adverse effect following GFS. Our explanation is in agreement with previous clinical study, showing that application of bevacizumab (a single VEGF inhibitor) and 5-FU (anti-metabolic drug) together during GFS significantly decreases failure rate after glaucoma surgery [25].

Trans-differentiation of HTFs to myofibroblasts and subsequent deposition of EMT are key steps during the scar formation following GFS [26]. In the process of EMT and fibrosis [27, 28], various cytokines and inflammatory factors (TGF, EGF, CTGF) act as molecular switches to start a series of downstream biological functions. EGFR, one of the tyrosine kinases (HER1-4) receptors, has a wide range of physiological functions in many activities of life [29]. Once EGFR binding with EGF, activated EGFR can trigger downstream signal pathways and ultimately elicit uncontrolled cell proliferation and fibrosis [29, 30]. ZD6474 can precisely target on EGFR to achieve its therapeutic effect. In the current study, we observed that higher expression of snail and α-SMA in TGF-β1-induced HTFs, but ZD6474 reversed the transformation from fibroblasts into myofibroblasts and EMT of TGF-β1-stimulated HTFs perhaps by inhibiting the activation of EGF to achieve the curative effect.

Conventional anti-metabolic drugs (MMC and 5‑FU) are routinely applied locally during and/or post operation (GFS) for inhibiting proliferation of fibroblasts in the wounded micro-environment. However, the major downside of MMC and 5‑FU is cytotoxicity, including bleb leakage, hypotony maculopathy, corneal toxicity and sclera melting [31, 32], compromising the application of MMC and 5‑FU significantly. In our current study, a transient 5-min exposure of MMC on HTFs could lead to mass cell death and apoptosis. By contrast, ZD6474 at the proper concentration was proved to be less toxic in HTFs. Therefore, ZD6474 had been confirmed with great curative effect and less side effects rather than MMC. Safety provides a strong guarantee for the follow-up research on ZD6474 to explore the curative effects.

Neovascularization, a major contributing factor for scaring formation, has been proved to be closely associated with fundus and glaucoma diseases [33, 34]. It is well documented that VEGF is secreted in both autocrine and paracrine fashions in the glaucomatous eyes at baseline, particularly over-secreted [5, 35, 36]. During the proliferative stage of wound healing, the overgrowth of angiogenesis results in new blood vessels formation with release of inflammatory molecular and cytokines that lead to scar formation and bleb dysfunction [35, 36]. As for the anti-neovascularization in our study, the proliferation and migration of HUVECs and tube formation were notably inhibited in the presence of ZD6474, suggesting that ZD6474 can robustly suppress neovascularization. Our finding is consistent with others, showing that ZD6474 significantly inhibits neovascularization to achieve anti-tumor effect in thyroid and breast cancers [18, 37]. Thus, our finding further suggests that ZD6474 seems to be a good candidate in reducing re-closure of pathway in trabecular meshwork by interfering formation of neovascularization post-GFS, i.e. anti-proliferation, anti-fibrotic and anti-angiogenesis, but substantially reduced adverse effect following GFS effectively.

Our western blot data showed that AKT-mTOR signaling pathway was downregulated in HTFs in response to ZD6474 treatment, suggesting that ZD6474 prevents scarring after GFS via reducing aqueous outflow resistance, using AKT-mTOR signaling pathway. This is in line with the finding that, mTOR inhibitor abates TGF‑β2‑induced fibrotic changes in trabecular meshwork cells and increases aqueous outflow in glaucoma treatment [38]. ZD6474 can accurately downregulate the downstream EGFR signaling pathway, which is at the heart of cell proliferation and migration [30], so targeting on EGFR to suppress its downstream network is widely exploited in fibrosis and EMT process [39, 40]. EGFR can recruit the PI3K lipid kinase via GAB1 and GAB2, and then PI3K catalyzes PIP2 into PIP3, which recruits AKT, leading to the activation of the PI3K -AKT-mTOR signaling pathway for various physiological functions [41]. Phosphorylation of AKT eventually leads to the inhibition of antagonists and cell proliferation and division [42]. Herein, EGF is closely related to the activation of AKT-mTOR signaling pathway. In present study, EGF-induced AKT-mTOR activation were considerably inhibited by ZD6474 treatment, as was EGF-induced EGFR phosphorylation.

VEGF, the most important angiogenesis factor, can be activated by binding with VEGF, and then many downstream signal-transduction networks associated with neovascularization can be successively activated, such as PI3K/AKT/mTOR, ERK1/2, and many others, so VEGF is crucial for angiogenesis-related processes [43]. In several previous studies, VEGF activation is confirmed to be tightly associated with the activation of PI3K-AKT-mTOR signaling pathway [44, 45]. To explore the possible mechanism of ZD6474 on HUVECs, p-AKT and p-mTOR expression were detected and found were obviously diminished. Hence, it is suggested that ZD6474 attenuates AKT/mTOR phosphorylation probably through a reduction in VEGF-receptor activation in HUVECs, resulting to the reduce of angiogenesis to prevent surgery failure after GFS.

Integrated with anti-fibrosis and anti-angiogenesis effects of ZD6474, we found that ZD6474 can play a positive role in preventing bleb dysfunction after GFS. Considering the low toxicity and good efficacy, ZD6474 can undoubtedly obtain great outcomes and applications in clinical. Hence, the present study implicates that multi-kinase inhibitors may improve outcomes with good safety during the period of postoperative recurrence after GFS.

It should be stated that there are some limitations in the present study. In vitro assays are not ample to assess the anti-fibrotic and anti-angiogenesis effect of ZD6474. Thus, it cannot be extrapolated directly in clinical situations. Additionally, the mechanism underlying the anti-fibrosis and anti-angiogenesis effects of ZD6474 warrants further investigation in animal model and in human glaucoma patients.

## Conclusion

In summary, ZD6474 treated on TGF-β1-stimulated HTFs inhibited cell migration, proliferation in HTFs, and attenuated trans-differentiation of HTFs to myofibroblasts. ZD6474 restrained tube formation and attenuate cell proliferation and migration in HUVECs, perhaps via AKT-mTOR signaling pathway in TGF‑β1‑stimulated HTFs and HUVECs. Our data strongly support that ZD6474 is a potential agent for prevention bleb dysfunction following GFS.

## Data Availability

The datasets used and/or analyzed during the present study are available from the authors on reasonable request.

## Abbreviations

BSA: Bovine serum albumin
CCK-8: Cell counting kit‑8
DMEM: Dulbecco’s modified Eagle medium
ECM: Endothelial cell medium
EGF: Epidermal growth factor
EGFR: Epidermal growth factor receptor
EMT: Epithelial‑mesenchymal transition
FBS: Fetal bovine serum
GFS: Glaucoma filtering surgery
HTFs: Human Tenon fibroblasts
HUVECs: Human umbilical vein endothelial cells
IER: Institute for eye research
MMC: Mitomycin C
*α*-SMA: *α*-Smooth muscle actin
TGF-*β*: Transforming growth factor *β*
VEGF: Vascular endothelial growth factor
VEGFR: Vascular endothelial growth factor receptor
